# Avian *Plasmodium* in *Culex* and *Ochlerotatus* Mosquitoes from Southern Spain: Effects of Season and Host-Feeding Source on Parasite Dynamics

**DOI:** 10.1371/journal.pone.0066237

**Published:** 2013-06-18

**Authors:** Martina Ferraguti, Josué Martínez-de la Puente, Joaquín Muñoz, David Roiz, Santiago Ruiz, Ramón Soriguer, Jordi Figuerola

**Affiliations:** 1 Departamento de Ecología de Humedales, Estación Biológica de Doñana (EBD-CSIC), Seville, Spain; 2 Servicio de Control de Mosquitos, Diputación de Huelva, Huelva, Spain; 3 Departamento de Etología y Conservación de la Biodiversidad, Estación Biológica de Doñana (EBD-CSIC), Seville, Spain; 4 Department of Biology, University of Oklahoma Biological Station, Kingston, Oklahoma, United States of America; Institut Pasteur, France

## Abstract

Haemosporidians, a group of vector-borne parasites that include *Plasmodium*, infect vertebrates including birds. Although mosquitoes are crucial elements in the transmission of avian malaria parasites, little is known of their ecology as vectors. We examined the presence of *Plasmodium* and *Haemoproteus* lineages in five mosquito species belonging to the genera *Culex* and *Ochlerotatus* to test for the effect of vector species, season and host-feeding source on the transmission dynamics of these pathogens. We analyzed 166 blood-fed individually and 5,579 unfed mosquitoes (grouped in 197 pools) from a locality in southern Spain. In all, 15 *Plasmodium* and two *Haemoproteus* lineages were identified on the basis of a fragment of 478 bp of the mitochondrial cytochrome *b* gene. Infection prevalence of blood parasites in unfed mosquitoes varied between species (range: 0–3.2%) and seasons. The feeding source was identified in 91 mosquitoes where 78% were identified as bird. We found that i) several *Plasmodium* lineages are shared among different *Culex* species and one *Plasmodium* lineage is shared between *Culex* and *Ochlerotatus* genera; ii) mosquitoes harboured *Haemoproteus* parasites; iii) pools of unfed females of mostly ornithophilic *Culex* species had a higher *Plasmodium* prevalence than the only mammophylic *Culex* species studied. However, the mammophylic *Ochlerotatus caspius* had in pool samples the greatest *Plasmodium* prevalence. This relative high prevalence may be determined by inter-specific differences in vector survival, susceptibility to infection but also the possibility that this species feeds on birds more frequently than previously thought. Finally, iv) infection rate of mosquitoes varies between seasons and reaches its maximum prevalence during autumn and minimum prevalence in spring.

## Introduction

Pathogens are selective factors for micro-evolutionary changes in their hosts and as such play an important role in regulating population dynamics [1]. Haemosporidian blood parasites include three main genera, *Plasmodium, Haemoproteus* and *Leucocytozoon*, all of which are phylogenetically closely related and have similar life cycles, but different life-history traits. Their life cycles require the intervention of insect vectors during sexual and sporogonic phases, along with a vertebrate host for the merogony phase and the development of gametocytes [2]. In birds, for an effective and successful transmission, a vector must first feed on an infected host to become infectious, and then this infectious vector must feed on another susceptible host [2]. Based on differences in suitable vectors and epidemiology, only species from the genus *Plasmodium* can be considered as malarial parasites [3]. However, other authors suggest that blood parasites belonging to *Haemoproteus* could be also referred as “malaria parasites” [4]. In particular, although it is closely related to mammal malaria, avian *Plasmodium* form a phylogenetically independent clade [5]. Nevertheless, despite their high diversity and widespread distribution range [2], [6], and contrary to the case of human malaria parasites, little information is available about the population dynamics of avian malaria parasites in natural ecosystems.

Although the interaction between blood parasites and birds has received considerable attention, the role of mosquitoes (Diptera: Culicidae) on parasite transmission has been comparatively less studied in the wild. This is probably due to the fact that, until the recent development of molecular tools, there were many difficulties in the detection and identification of the particular parasite lineages infecting mosquitoes. These molecular techniques have significantly improved our capacity for characterising the networks of avian blood parasite transmission [7]–[9]. However, information regarding the interaction between potential vectors and avian haemosporidians is still scarce.

The complex interaction between blood parasites and their vectors can be affected by both genetic and ecological factors [10], which can cause spatial and temporal variation in the parasite’s prevalence throughout its distribution [11]–[13]. The transmission dynamics of vector-borne pathogens can be affected by the population dynamics of mosquitoes [13], [14] and the density of susceptible hosts [15], as well as the environmental factors, for example weather conditions [16], potentially affecting both vector behaviour [17], [18] and the parasite development in their hosts [19].

Based on human vector-borne disease studies and pathogen models (such as *Plasmodium* or West Nile virus), three main factors are supposed to affect the prevalence of pathogens in mosquitoes [20], 21. First, different mosquito species – and even populations – have different susceptibilities to infection and capacities for transmitting parasites [15], [22]; second, the mosquito’s lifespan influences the likelihood of transmission to new hosts [23]; and, finally, the mosquito’s feeding behaviour determines contact rates with infected and susceptible vertebrates [24], [25]. Furthermore, some studies suggest that *Plasmodium* may alter the feeding preferences [26] and feeding behaviour [27] of infected mosquitoes.

Here, we studied the interaction between avian *Plasmodium* and *Haemoproteus* parasites with different mosquito species to assess: i) the potential relative importance of each mosquito species on the circulation of parasite lineages and ii) the influence of mosquito species, season and host feeding-source in the dynamics of avian malaria parasites in a mosquito community from a wetland area close to the Doñana National Park (Southwest Spain). At an inter-specific level, we expected that the mosquito species feeding mainly on birds would be more exposed to infection by avian *Plasmodium* and would have higher prevalence than species feeding mainly on mammals.

## Materials and Methods

### Study Area and Sample Collection

Mosquitoes were captured from February to November 2009 in the Cañada de los Pájaros (Seville, Spain; 6°14′W, 36°57′N), a freshwater lake (ca. 5 ha) surrounded by rice fields that originated from the restoration of an abandoned gravel pit. The study area is located approximately 20 km from the natural wetlands of Doñana National Park, an important bird stopover wetland in southwest Europe that hosts a high number of vertebrate species [28] and where a great diversity of mosquito species occurs in this area [29].

We trapped mosquitoes using Center for Disease Control (CDC) incandescent light-traps baited with dry ice as source of CO_2_, which were operational for 24 hours once or twice a week. Mosquitoes were also captured with a CDC backpack aspirator, model 2846. Trapping of mosquitoes was done with all the necessary permits from landowners and regional Department of the Environment (Consejería de Medio Ambiente, Junta de Andalucía). Samples were preserved in dry ice and then transported to the laboratory. Frozen mosquitoes were placed on a piece of filter paper in a Petri dish on a chill table, separated by gender and feeding status, and identified under a stereo microscope to species level using appropriate taxonomic keys [30], [31]. *Culex* mosquitoes belonging to the *univittatus* complex were identified as *Cx. perexiguus* based on the criteria detailed in Harbach [32].

Blood-fed females were identified visually by their dilated red abdomens and stored individually at −20°C until subsequent bloodmeal analyses. Unfed females were grouped in pools containing from 1 to 50 mosquitoes according to species and date of collection. Each pool was homogenized in a range of 500–700 µl of minimal essential medium (MEM solution) supplemented with 200 U/ml of antimicrobial drugs (penicillin/streptomycin) and 10% of fetal bovine serum and then stored at –80°C for subsequent analyses.

### Molecular Detection of Blood Parasites

The source of the bloodmeal (i.e., abdomen content) in our samples has been previously reported in Muñoz *et al*. [25] (see results). Here, we isolated genomic DNA using the remaining head-thorax of each blood-fed specimen to identify the presence of blood parasites in the mosquito. The head-thorax was ground with a sterilized pestle in vials containing 300 µl of SET buffer (100 mM NaCl, 50 mM Tris-HCl, pH 8, 50 mM EDTA, pH 8.0, SDS 1%), 5 µl proteinase K (20 mg/ml) and 10 µl DDT (1 M), and subsequently maintained overnight in an incubating shaker at 55°C. Once the digestion was completed, an equal volume (300 µl) of 5 M LiCl was added to each tube and each sample was mixed thoroughly by inversion with the addition of 630 µl of chloroform-isoamyl alcohol (24∶1). Samples were spun and the supernatant (around 500 µl) was carefully transferred to a new tube and the DNA precipitated with absolute ethanol. After recovery by centrifugation, the DNA was dried and washed with 70% ethanol and the final pellet was recovered and stored in water (see [33]). DNA extracts were checked by running 5 µl on a 0.8% agarose gel. In the case of unfed mosquitoes in the pools, the same protocol was used to isolate genomic DNA from 100 µl of the MEM solution containing the mosquitoes.

A 478 bp fragment (excluding primers) of the *Plasmodium* and *Haemoproteus* cytochrome *b* gen (cyt *b*) was amplified following the instructions provided by Hellgren *et al*. [34]. We assumed that false positives were negligible due to the strict laboratory protocols and the fact that negative controls for genomic DNA extraction and PCR (at least one per plate) showed no contamination. For negative samples in a first screening, we repeated the complete PCR protocol at least twice per sample to avoid false negative samples. When the probability of false positives is low and the true detection probability is at least 50%, it is recommendable to repeat each negative sample at least two times in order to significantly reduce bias in prevalence estimates [35].

Sequencing reactions were performed according to Big Dye 1.1 technology (Applied Biosystems). Labelled DNA fragments of PCR positive products were resolved with an ABI 3130xl automated sequencer (Applied Biosystems) using the same forward and reverse primers as used in the PCR reaction. Sequences were edited using the software Sequencher™ v4.9 (Gene Codes Corp., © 1991–2009, Ann Arbor, MI 48108) and identified by comparison with the GenBank DNA sequence database (National Center for Biotechnology Information, Blast, 2008) to assign unknown cyt *b* sequences to previously identified parasite lineages.

### Phylogenetic and Statistical Analyses

To investigate the phylogenetic relationships of the lineages we compared sequences obtained in this study to 32 *Plasmodium* lineages of known morphospecies previously deposited in MalAvi [6] and GenBank databases. The sequences were aligned using the CLUSTALW algorithm implemented in MEGA5 [36], and a fragment length of 478 pb was chosen for further analyses and comparison with previous blood parasite lineages. Based on results from the preliminary phylogenetic analysis including sequences from all the 32 morphospecies, we selected, based on bootstrap values (all ≥95%) and genetic distances lower than 5%, those *Plasmodium* lineages phylogenetically related with lineages isolated in this study. Genetic distances among distinguishable morphospecies are usually ≥5% for cyt *b* sequences [37]–[38]. However, there are many exceptions with distinguishable morphospecies with genetic divergence <1% [39]–[40]. Consequently, the clusters we identify should be considered as an instrument to group the different lineages instead of proof of morphological species identity. Only the two *Haemoproteus* sequences isolated in this study were included in the analyses as they had a 100% overlap with sequences of previously identified morphospecies. Finally, phylogenetic analyses were carried out using the Maximum Likelihood method based on the specific parameters for a model GTR+G+I (G-value = 0.5140; I-value = 0.3220) suggested by jModelTest 2 (Akaike Information Criterion) [40]–[42]. Nodal support was estimated by bootstrap analysis with 10,000 replications. Two sequences of *Leucocytozoon* corresponding to lineages GRUS1 (DQ847257) and SYAT2 (DQ847235) were used as out-groups.

The prevalence of blood parasites in mosquitoes was estimated separately for blood-fed and unfed mosquitoes. Parasite prevalence in blood-fed mosquitoes was calculated as the number of infected mosquitoes per the total of blood-fed mosquitoes tested for each mosquito species. In the case of unfed mosquitoes, parasite prevalence was estimated using *EpiTools* software available from AusVet Animal Health Services, Australia [http://epitools.ausvet.com.au/content.php?page=home]. This algorithm estimates the prevalence of infection and confidence limits from pooled samples taking into account differences in pool size and assuming 100% sensitivity and specificity [43].

The relationships between season and the prevalence of infection in different mosquito species were tested using the GENMOD procedure in SAS 9.2 software (SAS-Institute, Cary, NC, USA). We fitted generalized linear models (GLM) with logit link and binomial error distribution separately for both individual blood-fed mosquitoes and unfed mosquitoes in pools. In these models, we included parasite infection status (infected or uninfected) as the dependent variable and mosquito species and season (spring: 21 March–21 June, summer: 22 June–22 September and autumn: 23 September–21 December) as factors. The number of mosquitoes per pool was also included as a co-variable in the analysis of the pools. Data from winter (from 22 December–20 March) were not included in these analyses owing to the small sample size.

## Results

### Mosquito Species Composition, Phenology and Blood Parasite Identification

**Figure 1:**
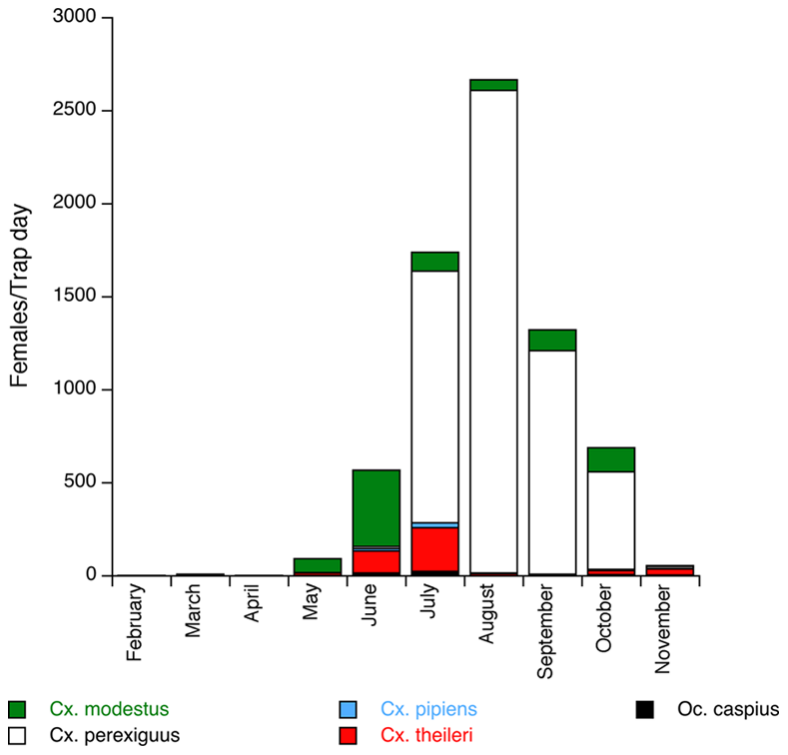
Mean number of female mosquitoes captured per day of trapping for the five species screened for blood parasites.

During 75 trapping sessions, we collected 58,138 female mosquitoes from nine different species: 45,573 *Culex perexiguus*, 7,094 *Cx. modestus*, 3,706 *Cx. theileri*, 728 *Anopheles atroparvus*, 556 *Ochlerotatus caspius*, 474 *Cx. pipiens*, 3 *Oc. detritus*, 2 *An. algeriensis* and 2 *Urotaenia unguiculata*. Mosquito abundance was low from February to April and increased to a maximum in August (see Figure 1). Abundances remained high in September and October, but dropped dramatically in November.

Due to logistical limitations only a subsample of all the captured unfed mosquitoes was analyzed for the presence of parasite. Analysed samples were stratified by month to cover as much as possible the phenology of each mosquito species. Specifically, the examined mosquitoes included 5,745 females belonging to the genera *Culex* and *Ochlerotatus*: *Cx. perexiguus* (*n = *2,216), *Cx. modestus* (*n = *1,790), *Cx. theileri* (*n* = 1,473), *Oc. caspius* (*n = *169) and *Cx. pipiens* (*n = *97).

Fifteen *Plasmodium* and two *Haemoproteus* lineages were identified. Eleven *Plasmodium* and two *Haemoproteus* lineages were isolated only from *Culex*, three *Plasmodium* lineages were exclusively found in *Ochlerotatus* and a single *Plasmodium* lineage was shared by both genera. Seven lineages completely matched with previously described lineages (sequences published in GenBank database): Rinshi-1 (*Plasmodium relictum*), Rinshi-11 (*P. vaughani*), pSPHUjJ, Delurb5, Yacho-1, PADOM05 (*Haemoproteus passeris*) and SYBOR1 (*H. parabelopolskyi*). Sequences from the ten new lineages of *Plasmodium* found in this study (Donana01 to Donana10, Table 1) were deposited in GenBank database (Table 2).

**Table 1:** Avian *Plasmodium* and *Haemoproteus* lineages detected in female blood-fed individuals and unfed mosquito pools.

		Mosquito species											
	Parasite lineages	Blood-fed mosquitoes				Unfed mosquito pools							
		*Cx. perexiguus*	*Cx. modestus*	*Cx. theileri*	Total	*Cx. perexiguus*	*Cx. modestus*		*Cx. pipiens*	*Oc. caspius*	Total		
*Plasmodium*	Delurb5			1	1	1					1		
	pSPHUjJ	3			3	2	1		1	1	5		
	Rinshi-1	9		2	11	7	3		2		12		
	Rinshi-11	4		1	5	3	1				4		
	Yacho-1	1			1						0		
	Donana01				0	1					1		
	Donana02				0		2				2		
	Donana03		1		1						0		
	Donana04				0		1				1		
	Donana05	1			1	1					1		
	Donana06				0					1	1		
	Donana07	1			1						0		
	Donana08				0					1	1		
	Donana09				0					1	1		
	Donana10				0	1					1		
*Haemoproteus*	PADOM05	1			1	1		1				2	
	SYBOR1	2		1	3							0	
	Unidentified co-infections	3	1	1	5					1		1	
	Total	25	2	6	33	17		9	3	5		34	

No infected blood-fed *Cx. pipiens* and unfed *Cx. theileri* were captured. Note that 31 infected pools were found with a total of 34 identified lineages (see results).

**Table 2:** *Plasmodium* and *Haemoproteus* lineages isolated in this study.

	Name	GenBank N°	Vector Species	Morphospecies	Continent	Host Order (Families)	Mosquito species of this study
***Plasmodium***	Delurb5	EU154347			Europe	Passeriformes (4)	*Cx. perexiguus, Cx. theileri*
	pSPHUjJ	AB604303			Asia	Sphenisciformes (1)	*Cx. pipiens, Cx. perexiguus,*
							*Cx. modestus, Oc. caspius*
	Rinshi-1	AB458849	*Cx. pipiens pallens, Cx. sasai*	*P. relictum*	Africa	Ciconiiformes (1)	*Oc. caspius, Cx. perexiguus, Cx. pipiens*
	( = SGS1)	AF495571			Asia	Galliformes (2)	*Cx. modestus, Cx. theileri*
	( = P22)	DQ659562			Europe	Gruiformes (1)	
	( = P22.3)	DQ839049				Passeriformes (14)	
						Procellariiformes (1)	
						Sphenisciformes (1)	
	Rinshi-11	AB477124	*Cx. pipiens pallens, Cx. restuans*	*P. vaughani*	America	Passeriformes (7)	*Cx. perexiguus, Cx. modestus, Cx. theileri*
	( = SYAT05)	DQ847271			Asia	Columbiformes (1)	
					Europe		
					Oceania		
	Yacho-1	AB477128	*Cx. pipiens pallens*		Asia		*Cx. perexiguus*
	( = CXPIP10)						
	Donana01	JX458326			Europe		*Cx. perexiguus*
	Donana02	JX458327			Europe		*Cx. modestus*
	Donana03	JX458328			Europe		*Cx. modestus*
	Donana04	JX975223			Europe		*Cx. modestus*
	Donana05	JX458329			Europe		*Cx. perexiguus*
	Donana06	JX458331			Europe		*Oc. caspius*
	Donana07	JX458332			Europe		*Cx. perexiguus*
	Donana08	JX458330			Europe		*Oc. caspius*
	Donana09	JX458333			Europe		*Oc. caspius*
	Donana10	JX975222			Europe		*Cx. perexiguus*
***Haemoproteus***	PADOM05	HM146898	*Cx. pipiens pallens*	*H. passeris*	Asia	Passeriformes (1)	*Cx. perexiguus, Cx. modestus*
					Europe		
	SYBOR01	AF495575		*H. parabelopolskyi*	Africa	Passeriformes (2)	*Cx. perexiguus, Cx. theileri*
					Asia		
					Europe		

GenBank accession numbers and corresponding species, as well as the vectors, bird species and locations from which they have been previously reported (see Bensch *et al*. [6], Santiago-Alarcon *et al.* [48] and GenBank databases). Number between parentheses in the Host Order column corresponds to the number of Families.

**Figure 2:**
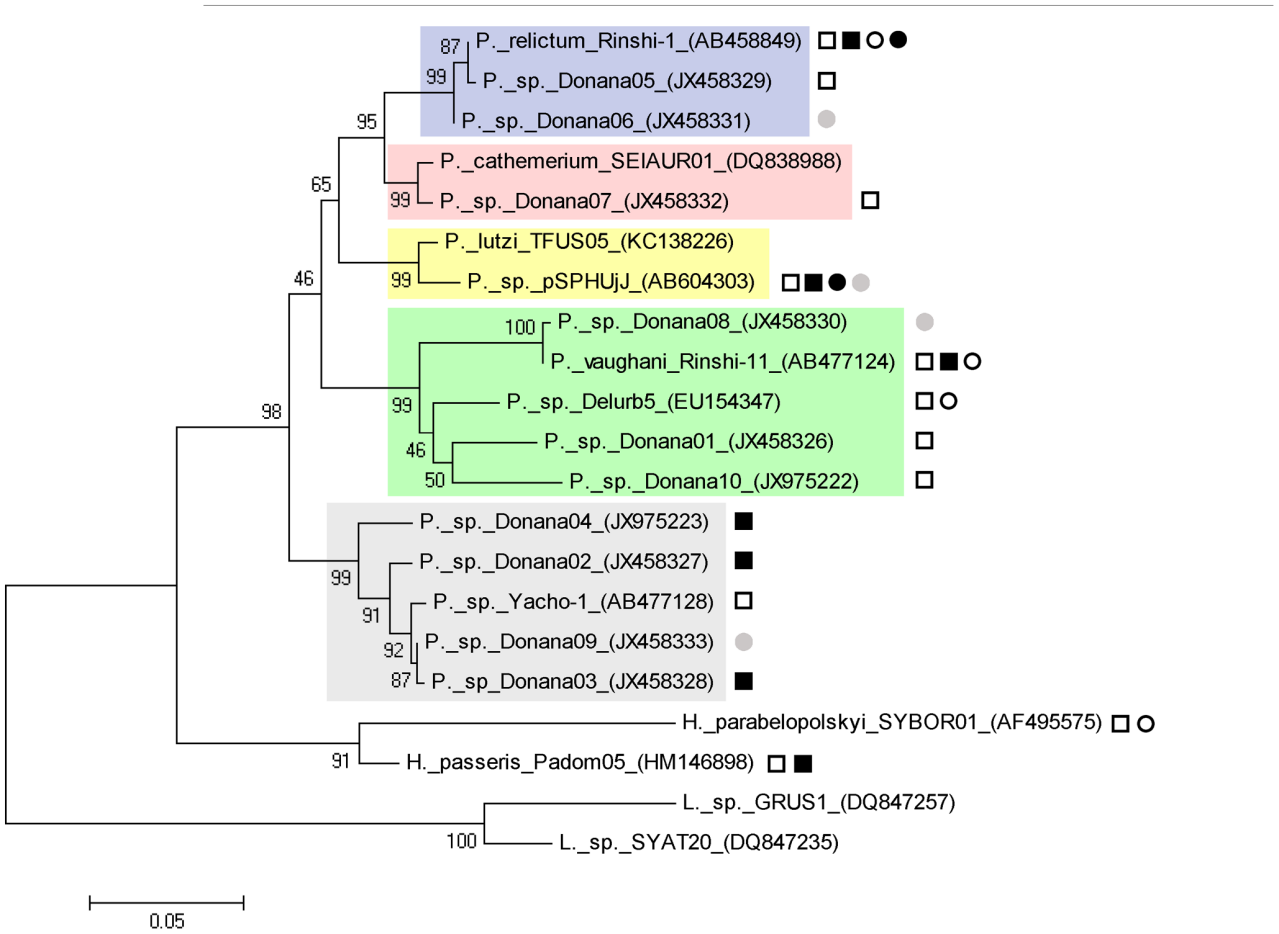
Phylogenetic relationships of *Plasmodium* and *Haemoproteus* lineages obtained in the current study based on cytochrome *b* sequences of 478 bp. Similar sequences from samples identified to morphospecies level available in GenBank and MalAvi were included for reference. *Leucocytozoon* GRUS1 and SYAT20 lineages were used as out-groups. The numbers on the top of the branches indicate bootstrap support (10 000 replications). The mosquito species infected by each parasite are represented as follows: *Cx. pipiens* (black circle); *Cx*. *theileri* (white circle); *Oc*. *caspius* (grey circle); *Cx*. *modestus* (black square); *Cx*. *perexiguus* (white square).

The phylogenetic tree including a total of 17 *Plasmodium*, two *Haemoproteus* and two *Leucocytozoon* lineages (out-groups), showed five highly supported (bootstrap value = 99%) clusters of *Plasmodium* lineages (Figure 2). A first cluster grouped Rinshi-1 lineage from *P. relictum* with Donana05 and Donana06, the last two genetically differing less than 0.4% with sequence from *P. relictum.* Lineages Donana07 and pSPHUjJ were grouped with sequences from *P. cathemerium* (genetic distance = 0.8%) and *P. lutzi* (genetic distance = 1.7%) respectively. Furthermore, a cluster grouped lineages Donana08, Donana01, Delurb5 and Donana10 with sequence Rinshi-11 from *P. vaughani*. In this case, Donana08 and Delurb5 differed respectively by 0.2% and 4.7% from *P. vaughani* sequence, while the rest of lineages showed considerably higher differences with this species (genetic distances >5%). Finally, there is a cluster of 4 lineages (Donana02, Donana03, Donana04 and Donana09) that were closely associated (less than 3.4% divergence) with the previously described Yacho-1 lineage, although there is no information available to assign these lineages to a particular morphospecies identity (Figure 2).

### Blood Parasites in Unfed Mosquito Pools

In all, 197 pools containing 5,579 mosquitoes of the species *Cx. pipiens, Cx. perexiguus, Cx. modestus, Cx. theileri* and *Oc. caspius* were screened for the presence of blood parasites; of these pools, 31 (15.7%) were positive. Three pools of 50 mosquitoes (one pool of *Cx. modestus* and two pools of *Cx. perexiguus*) were infected simultaneously by two different lineages which were individually identified in different PCR reactions. Another pool showed evidence of coinfection but the parasites could not be identified to lineage level. Parasite prevalence for each mosquito species is shown in Table 3. Despite the existence of techniques for resolving double lineages through TA-cloning [44] or the design of new set of specific primers [45], these methods were not used in this study due to the small number of coinfections. In total, 13 different lineages were found in unfed mosquito pools; lineages Rinshi-1, pSPHUjJ and Rinshi-11 were the most prevalent. The other parasite lineages were isolated only from one or two pools (Table 1).

**Table 3:** Prevalence of avian malaria parasites in unfed mosquitoes.

Mosquito species	N° Pools	Mean	Range	Positive pools	Prevalence (%)	Lower 95% CL	Upper 95% CL
*Cx. modestus*	49	35.857	1–50	8	0.5	0.2	0.9
*Cx. pipiens*	24	3.958	1–27	3	3.2	0.8	8.2
*Cx. perexiguus*	50	42.340	1–50	15	0.8	0.4	1.3
*Cx. theileri*	51	28.244	1–50	0	0.0	0.0	0.0
*Oc. caspius*	23	7.348	1–28	5	3.1	1.1	6.6

Prevalence and confidence limits estimated with *EpiTools*.

### Blood Parasites in Blood-fed Mosquitoes

In total, 33 of 166 (19.9%) blood-fed mosquitoes had a positive amplification of malaria parasites. The parasite prevalence was highest in *Cx. perexiguus* (n = 100; prevalence = 25.0%), followed by *Cx. theileri* (32; 18.8%), *Cx. modestus* (32; 6.3%) and *Cx. pipiens* (2; 0%).

We isolated 10 different parasite lineages from these mosquitoes, with a predominance of Rinshi-1, followed by Rinshi-11 and SYBOR1. The remaining seven parasite lineages were isolated from only one or two mosquitoes (Table 1). In five mosquitoes, we found evidence of co-infection and parasite lineages were not identified.

The feeding source was only identified in the case of 91 mosquitoes (out of 166 analyzed). The origin of the bloodmeals for each species was as follows: *Cx. perexiguus* –45 birds and 10 mammals; *Cx. modestus* –15 birds, 1 mammal and 1 reptile; *Cx. theileri* –10 birds and 8 mammals; and *Cx. pipiens* –1 bird. Twelve (17.6%) and four (18.2%) head-thoraxes from blood-fed mosquitoes containing an avian or mammal-derived bloodmeal in their abdomen, respectively, were positive.

### Temporal Dynamics of Parasite Transmission

**Figure 3:**
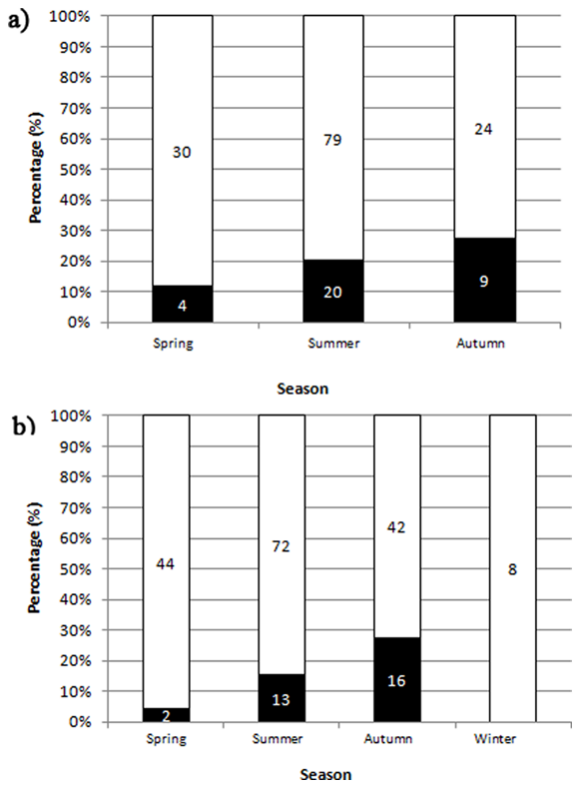
Seasonal pattern of blood parasite in the studied area. Numbers in bars indicate sample size. (A) Percentage of infected (black) and uninfected (white) blood-fed mosquitoes. Note: No blood-fed mosquitoes were captured in winter. (B) Percentage of pools containing infected (black) and uninfected (white) unfed mosquitoes. Note: for the case of mosquitoes in pools, figure show proportions of infected pools but not parasite prevalence. The seasonal infection prevalence (95% coeficient intervals) for mosquitoes in pools were: Spring = 0.56% (0.09–1.74), Summer = 0.99% (0.55–1.64), Autumn = 2.54% (1.47–4.06).

We tested the effects of seasonality on the rate of infection of both blood-fed and unfed pooled mosquitoes. Despite the fact that the abundance of mosquitoes peaked in summer (blood-fed mosquitoes were not captured in winter), the prevalence of infection increased as the year progressed, reaching a maximum in autumn (Figure 3). Eight unfed mosquito pools collected in February and March were not infected. No mosquitoes were collected in January and December.

For the case of unfed mosquitoes in pools, a significant effect of both mosquito species (df = 4; χ^2^ = 23.68; *p*<0.0001) and season (df = 2; χ^2^ = 9.83; *p* = 0.0074) on the infection rate was found after controlling for the non-significant effect of the number of mosquitoes per pool (df = 1; χ^2^ = 0.01; *p* = 0.92). Post-hoc analyses revealed that *Cx. theileri* showed a significant lower proportion of infected pools than the rest of species (all *p*<0.006). Furthermore, a higher proportion of positive pools were found in autumn than in spring (*p* = 0.002); in addition, the proportion of infected pools tended to differ between autumn and summer (*p* = 0.07) and between spring and summer (*p* = 0.07) (Figure 3). For the case of blood-fed mosquitoes, we did not find significant effect for mosquito species (df = 3; χ^2^ = 6.06; *p* = 0.11) or season (Figure 3; df = 2; χ^2^ = 1.44; *p* = 0.49) on the proportion of infected females.

## Discussion

The identification of potential vectors is an essential step in transmission studies of vector-borne diseases. To our knowledge, this is the first molecular detection and characterization of avian *Plasmodium* and *Haemoproteus* lineages from mosquitoes in Spain. Parasite prevalence varied between species in agreement with findings from other studies [15], [46], [47]. Although the four *Culex* species tested here, *Cx. modestus, Cx. perexiguus, Cx. pipiens* and *Cx. theileri* have been previously reported as potential vectors of avian *Plasmodium* (see Table 3 and ref. [48], [49]), this is the first report for *Oc. caspius* as a potential host of avian malaria. Both *Cx. theileri* and *Oc. caspius* are presumed to have a preference for biting on mammals [25], [50], [51] and so we expected a lower prevalence of infection by avian malaria parasites than in species that mainly feed on birds (i.e. *Cx. modestus*, *Cx. perexiguus* and *Cx. pipiens*). However, this is not the case as *Cx. theileri* blood-fed females had a parasite prevalence of almost 20%, and unfed *Oc. caspius* mosquitoes of 3.1%. The high infection rates in *Oc. caspius* may be related to the fact that this species is multivoltine, laying several successive clutches and feeding on multiple individual hosts during its life cycle. This fact together with the high diversity and abundance of avian species in the studied area could favour the potential infection by blood parasites of this species if it had fed, at least once, on an infected bird. Moreover it is also possible that *Oc. caspius* is more ornithophilic than previously assumed although previous studies suggest that this may be not the case ([25], [52], authors unpublished data). Also, it could be possible that differences between mosquito species in their capacity to be infected and/or to transmit pathogens could affect our results. In fact, these differences have been widely reported for viruses and protozoans [15], [22] but have rarely been quantitatively investigated for avian *Plasmodium* [15]. A greater survival rate in comparison to *Culex* species may also explain these differences. Moreover, it is possible that our prevalence estimation was biased due to a relatively low sample size (23 pools) for *Oc. caspius* included in this study [53].

When analyzing blood-fed mosquitoes, the prevalence of infection did not significantly differ among species. This lack of difference was probably due to the relative low sample size for some species, but also for the potential detection of pathogens in the vertebrate bloodmeal prior to successful infection of the mosquito by the parasite. Amplification of parasites present in the vertebrate blood but not in the mosquito, may also explain the higher prevalences of *Plasmodium* we found in blood-fed in comparison with unfed mosquitoes. If this is the case we expect to find a higher proportion of positives for *Plasmodium* in females that had feed on avian blood than in those that had feed on mammals, because the last are not infected by this group of *Plasmodium*. However the proportion of blood-fed females positive for *Plasmodium* was similar for mosquitoes with avian or mammal bloodmeal suggesting that amplification of *Plasmodium* from vertebrate blood had not highly biased our results. The more likely explanation for the lower *Plasmodium* prevalence among unfed females is the inclusion of a large proportion of nulliparous females that have never taken a bloodmeal and consequently could not have been infected by *Plasmodium*. The identification of the hosts in the 75 blood-fed individuals probably failed due to the advanced state of digestion of the bloodmeals and or the DNA extraction procedure employed (see ref. [25] for details, [54]). Consequently, this result should be considered with caution due to the relatively low sample size.

### *Plasmodium*-mosquito Interactions

Although some *Plasmodium* lineages are strict vector specialists [55], most *Plasmodium* parasites are probably able to use many vector species for transmission, including species other than Culicidae [46], [47], [56]. However, there is considerable variation in host breadth in numerous lineages [47], [57]. As support for this possibility, our results show that different mosquito species belonging to two different genera share closely related or almost identical *Plasmodium* lineages, thereby suggesting the existence of multiple vector-parasite assemblages (Table 1 and Figure 2; see also ref. [47], [48]). This may be the case above all for *P. relictum*, a generalist *Plasmodium* parasite isolated in this study (lineage Rinshi-1). This widespread parasite species is transmitted by at least 24 mosquito species [2], [48] and has been also isolated from four species in our study. However, specialist and generalist strategies might only be the extremes of a continuum. For example, the evolutionary relationships of avian blood parasites have revealed that several lineages of *Plasmodium* exhibit extreme generalist host–parasitism strategies, whereas other lineages appear to be restricted to certain host families or species [57]–[59]. Despite finding no strict associations between mosquito species and *Plasmodium* lineages, we cannot exclude the existence of quantitative differences in the parasite load among mosquito species. To test this idea it will be necessary to conduct further studies on the abundance of each parasite lineage infecting each mosquito species (e.g., using real time PCR) as a way of identifying not only the presence/absence of each parasite lineage, but also as a means of detecting potential quantitative differences in the intensity of infection by *Plasmodium.*

### *Haemoproteus* Isolation from Mosquitoes

One interesting finding of our study is the detection of *Haemoproteus* lineages in three mosquito species. It is generally assumed that the majority of *Haemoproteus* species are transmitted by biting midges belonging to the genus *Culicoides* [2]. However, a number of recent studies have reported the isolation of *Haemoproteus* lineages in mosquitoes [47], [60]–[62], although the identification of a parasite in an insect does not necessarily imply the existence of an infection by that parasite lineage nor the capacity to transmit it [63]. However, in our study two *Haemoproteus* lineages appear indiscriminately in blood-fed and unfed mosquitoes, including a *Haemoproteus* lineage isolated from one blood-fed mosquito (*Cx. perexiguus*) with rabbit blood (*Oryctolagus cuniculus*) in its abdomen. This finding suggests that the parasite infected the mosquito during a previous feed on an infected bird. Alternatively, this could be a case of double feeding (on a mammal and on a bird in a short time period), although this is doubtful because there is no evidence of double peaks in the sequences. Likewise, it would seem to be unlikely that we would have amplified DNA from a parasite that infects only a fraction of red blood cells, but not the DNA from the avian host present in the blood cells themselves. Our study, along with other previous reports [47], [60], [62], reveals the need to conduct further laboratory experiments on the capacity of mosquitoes to harbour and potentially transmit *Haemoproteus* parasites.

### Transmission Dynamics of Avian Malaria Blood Parasites

The transmission of vector-borne diseases depends on different factors including the relative size of the vector population, vector biting rates and parasite development within vectors [14], [64], [65]. In addition to seasonal changes in parasite transmission, periodical changes in host demographic rates and, especially, seasonal pulses of births, can expand or diminish the abundance and proportion of susceptible hosts with concomitant impacts on infection rates [19]. Given that some *Culex* species including *Cx. perexiguus, Cx. modestus* and *Cx. pipiens* fed primarily on birds [25], [50], [66] and the fact that the density of these mosquito species in our studied area peaked from July to September (Figure 1), these mosquito species have the potential to play an important role in parasite transmission during autumn and in the dynamics of avian malaria parasites in the bird community. Our results for the temporal pattern of infection are consistent with findings from the few previous studies of the temporal dynamics of avian blood parasites [9], [13], [62]. For example, Inci *et al*. [62] found that there was a greater prevalence of infection in mosquitoes captured in August than in June and July in Turkey. Kim *et al.* [9] found seasonal changes in composition of infected *Culex* species, with infection rates that increased continuously from July, when the first infected mosquito was captured, to October. In another study, also conducted in Japan, Kim & Tsuda [13] observed an increase in *Plasmodium* prevalence both in blood-fed and unfed *Culex* mosquitoes throughout the year, with a first peak in summer and another in autumn. As previously noted by these authors, the increase in the parasite prevalence during the year could be due to the density of host-seeking females and the infection rate of avian malaria parasites reaching during summer or autumn coinciding with the presence of a number of susceptible fledglings of resident birds and summer visitors and also to the fact that mosquito population age may increase along the seasons. Together, these factors may potentially favour the persistence of circulating parasites in mosquitoes after the decline in blood parasite infections in birds during reproduction.

In conclusion, we found that in Spain i) different *Plasmodium* lineages are shared among *Culex* species and one *Plasmodium* lineage is shared between *Culex* and *Ochlerotatus* genera*;* ii) mosquitoes could be implicated in the transmission of some *Haemoproteus* lineages, but this should be tested in the laboratory; iii) unfed females from ornithophilic *Culex* species had higher prevalence of *Plasmodium/Haemoproteus* than those of *Culex* species feeding mainly on mammals; and iv) the infection rate of mosquitoes increases among seasons, reaching its maximum prevalence in autumn.
